# Gold deposit type and implication for exploration in the Abiete-Toko Gold District, South Cameroon: constraint from morphology and microchemistry of alluvial gold grains

**DOI:** 10.1016/j.heliyon.2021.e06758

**Published:** 2021-04-09

**Authors:** Gus Djibril Kouankap Nono, Edelquine Fai Bongsiysi, Primus Azinwi Tamfuh, Alexis Jacob Nyangono Abolo, Bertrand Fomekong Kehding, Nicoline Fontem Kibong, Emmanuel Cheo Suh

**Affiliations:** a Department of Geology, Higher Teacher Training College, The University of Bamenda, P.O. Box 39 Bambili, Cameroon; b Department of Geology, Mining and Environmental Science, Faculty of Science, University of Bamenda, P.O. Box 39 Bambili, Cameroon; c Department of Mining and Mineral Engineering, National Higher Polytechnic Institute, University of Bamenda, P.O. Box 39 Bambili, Cameroon; d Department of Soil Science, Faculty of Agronomy and Agricultural Sciences, University of Dschang, P.O. Box 222 Dschang, Cameroon; e Department of Earth Science, Faculty of Science, University of Yaoundé 1, P.O. Box 812 Yaoundé, Cameroon; f Economic Geology Unit, Department of Geology and Environmental Science, University of Buea, P.O. Box 63 Buea, Cameroon

**Keywords:** Nyong series, Greenstones, Gold grain morphology, Microchemistry, Orogenic gold deposit and exploration

## Abstract

The morphology and quantitative chemical analyses of fifty alluvial gold grains from fourteen studied sites were used to constraint gold deposit type and its implications to exploration in the Abiete-Toko Gold District in South Cameroon. The main results revealed that the gold grains show a core-rim zonation marked by Ag depleted rims as a result of leaching during transportation. The fineness of grains ranges from 826 to 1000 and their composition is almost binary gold-silver. Gold fineness refers to the relative amounts of Ag and Au present, given as a number out of 1000 and defined by Au/(Ag + Au)∗1000. The chemical composition of the gold grains are range as thus: 83.40–100 wt.% Au, 0.07–17.45 wt.% Ag, 0–0.96 wt.% Cu, 0–0.01 wt.% As, 0–0.02 wt.% Ni, 0–0.02 wt.% Co, 0–0.01 wt.% Se, 0–0.08 wt.% Hg and 0.003–0.03 wt.% S. The high sulphur concentrations of the gold grains probably imply primary deposition of gold by sulphidation; The ranges of Ni and Co concentrations suggest an interaction with greenstones. Such findings indicate that the Abiete-Toko area hosts a mesothermal-orogenic gold deposit. Exploration operations must target fractures and contact zones between ironstones, felsic gneiss and greenstones. Soil sampling is not recommended in this orogenic gold district due to the very thick and transported nature of the weathering mantle. Detailed mapping coupled to geophysical surveys are the recommended key exploration methods prior to drilling program.

## 1 Introduction

**Figure 1:**
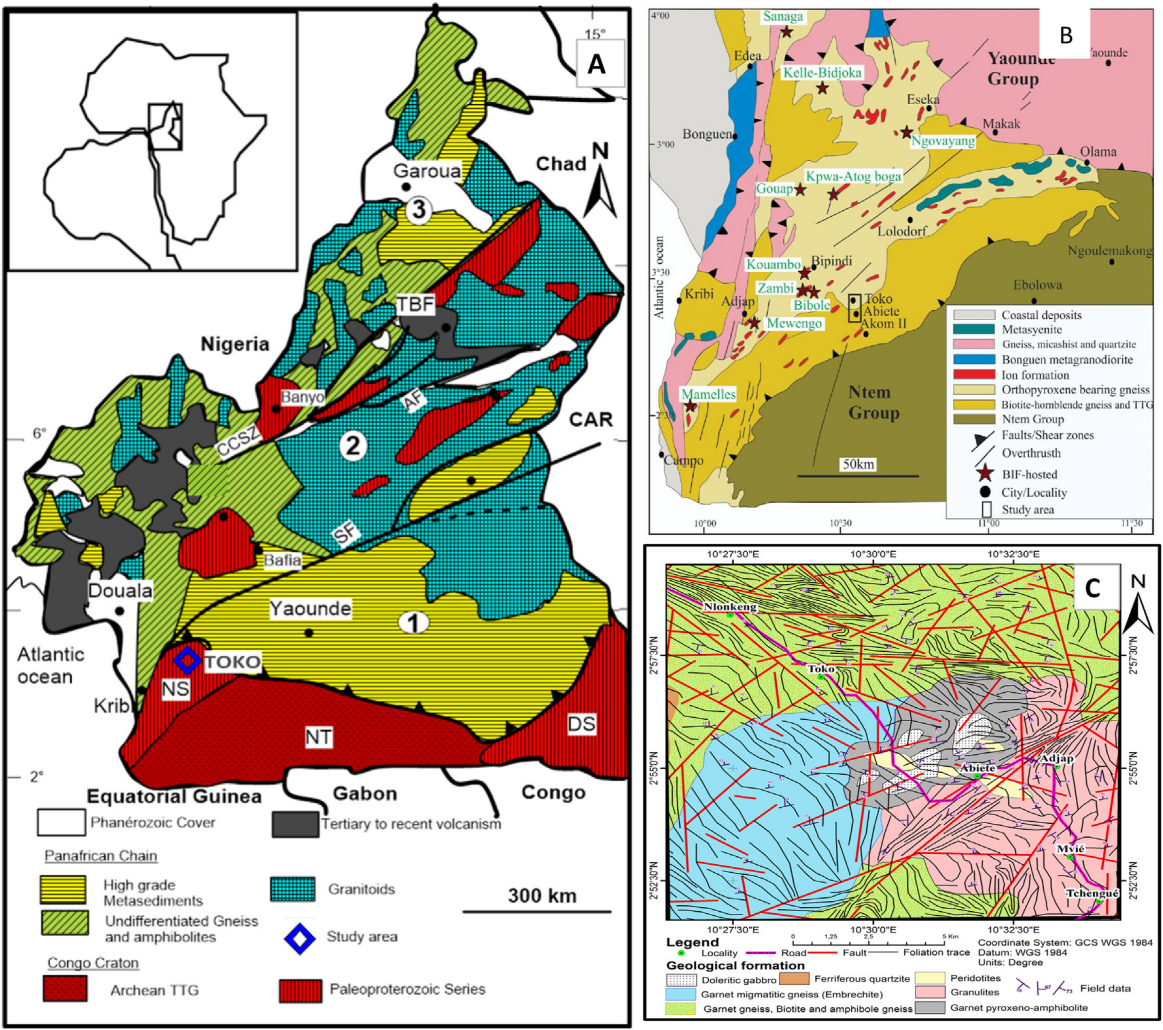
Geological maps; (A) Map of Cameroon (modified from Nzenti et al., 1994) showing the CCSZ (Central Cameroon Shear Zone); CAR (Central African Rift); AF (Adamawa Fault); SF (Sanaga Fault); TBF(Tibati-Banyo Fault); NT (Ntem complex); DS (Dja Series); NS (Nyong Series). (1) Southern Domain; (2) Central Domain and (3) Northern Domain. (B) Simplified geological map of Ntem Complex in the Cameroon section of the Congo Craton (modified from Nzenti et al., 1994); (C) Map of Abiete-Toko gold district after Binam Mandeng et al. (2018).

Most world class primary and secondary gold deposits occur within Precambrian basements mostly in greenstone belts (Groves et al., 1998; Pitfield et al., 2009; Zoheir, 2012). The Precambrian basement in Cameroon is divided into the Pan-African North Equatorial Fold Belt and the Ntem Complex (Figure 1A). The Ntem complex forms the Northern part of the Congo Craton in Cameroon and is subdivided into the Nyong series, the Ntem complex and the Ayna series (Nzenti et al., 1994, 1999; Lerouge et al., 2006; Pouclet et al., 2007; Mbongue et al., 2014; Moudioh et al., 2020). The Nyong Series, to which belongs the Abiete-Toko gold district, is a supercrustal formation that was emplaced during the Paleoproterozoic, metamorphosed at 2050 Ma during the Eburnean orogeny and later overprinted by Pan-African events (Lerouge et al., 2006; Ako et al., 2015; Moudioh et al., 2020). The Nyong series, affected by polyphasic deformation and dissected by the greenstone belt, consists of three rock units, which include: meta-sedimentary, meta-igneous and the syn-to late-tectonic granitoids and syenites (Aye et al., 2017; Binam Mandeng et al., 2018; Kankeu et al., 2018; Bouyo et al., 2019; Fuanya et al., 2019).

Important small scale alluvial gold mining sites in Cameroon are found in the Adamawa and East Regions, but other smaller active alluvial mining sites exist, among which is the Abiete-Toko Gold District where relatively little or no gold exploration has been undertaken (Embui et al., 2013; Soh et al., 2014; Vishiti et al., 2015, 2017). Moreover, geochemical dispersion of Gold grains has been carried out in the Nyong series and proven to be pure gold, not associated with impurities of chalcophile elements (Mimba et al., 2014). The morphology of gold grains evaluated along the Nyong River shows that they have variable shapes and sizes (Omang et al., 2015). Yet, no detailed study has been carried out in the Abiete-Toko Gold District concerning the morphology and microchemistry of gold grains. The Nlonkeng and Mvouba Rivers are the most artisanally acive gold mining sites in Abiete-Toko area for over 50 years. Mineral exploration has earlier been conducted in this area by AFFERO MINING LTD (TXE) and currently by STONES AND GOLD Sarl Mining Companies. The morphology of gold grains is a useful indicator of fluvial transport distance while gold microchemistry provides useful information on the mechanism of gold deposition (Chapman and Mortensen, 2006; Vishiti et al., 2017). Angular gold grains indicate short fluvial transport distance and rounded grains show long fluvial transport distance and this enables to determine the approximate area of the ore source. The implication of gold grains morphology in river channel demarcation is obvious in mineral exploration programs as it helps to extend or narrow the possible source area of gold exploration (Omang et al., 2015).

This paper presents the first data on morphology and microchemistry of gold grains from stream sediments in Abiete-Toko gold district, with the aim of shedding light on the gold grain morphology and composition, deposit types and the implications for gold exploration in the area.

## 2 Geographical and geological setting

### 2.1 Geographical setting of the Abiete-Toko Gold District

The Abiete-Toko gold district is situated 23 km north of Akom II Town (South Region of Cameroon). It covers a surface area of about 232 km^2^ and is located between latitude 2^o^55^’^48″N - 2^o^52^’^12″N and longitudes 10^o^27^’^0″E - 10^o^30^’^36″E (Figure 1).

The climate of Akom II is the equatorial type with four seasons: two rainy seasons and two dry seasons. The average monthly temperature is 24.3 °C and the annual rainfall is 2188 mm. The vegetation cover is the humid dense forest and the marshy vegetation (Letouzey, 1985). The relief of the Akom II is relatively rugged and the altitudes range between 72 m and 630 m. The drainage network is dense and dendritic with numerous waterfalls offering great hydroelectric and touristic potential. The major soil types are the red and yellow ferrallitic soil along the slopes while the valley bottoms are covered hydromorphic soils.

### 2.2 Geological setting

#### 2.2.1 Regional geology (The Nyong Series)

The Abiete-Toko gold district is located within the Nyong series in the North-West border of the Ntem Complex (Figure 1B). It lies on the Congo Craton as an Eburnian formation (Toteu et al., 1994; Feybesse et al., 1998). This series is a reactivated portion of the Ntem complex in Cameroon which consists of a Meta-sedimentary rock unit (garnet-bearing mica schist, mica schist and schist), the Meta-igneous rock unit (pyroxene-rich gneiss, garnet-rich charnockitic gneiss, charnockitic gneiss, amphibolite biotite-rich gneiss, and garnet-rich amphibolite) and the syn-to late-tectonic syenites and granitoids (Pouclet et al., 2007; Mbongue et al., 2014; Ngo Bidjeck, 2004; Aye et al., 2017; Bouyo et al., 2019; Fuanya et al., 2019). The Nyong unit is characterized by a D1-D3 polyphasic deformation (Binam Mandeng et al., 2018; Kankeu et al., 2018).

#### 2.2.2 Local geology and the structural settings of the Abiete-Toko area

The lithology of the Abiete-Toko gold district consists of Grt + Bt + Amp-bearing gneiss, Grt-bearing migmatite, granulite, Grt-bearing pyroxene-amphibolites, gabbro and peridotite (Binam Mandeng et al., 2018). Structurally, this precambian formations has been affected by three deformation phases (D1, D2 and D3) in line with the three deformation phases of the Nyong series. Remote sensing and field mapping of this areas have revaled a dextral WNW-ESE shear zone and a sinistral NE-SW shear zone suggesting a N–S and NE-SW shortening expressed as folds and localized strike-slip shear zones (Binam Mandeng et al., 2018). Mafic and ultramafic rock occurences in Abiete are possible sources of primary gold and related strategic metals (Ngo Bidjeck, 2004). Microchemical signature of alluvial gold from two contrasting terrains in Cameroon suggests an ultramafic source rock for the Nyong gold grains (Omang et al., 2015).

## 3 Methods

**Figure 2:**
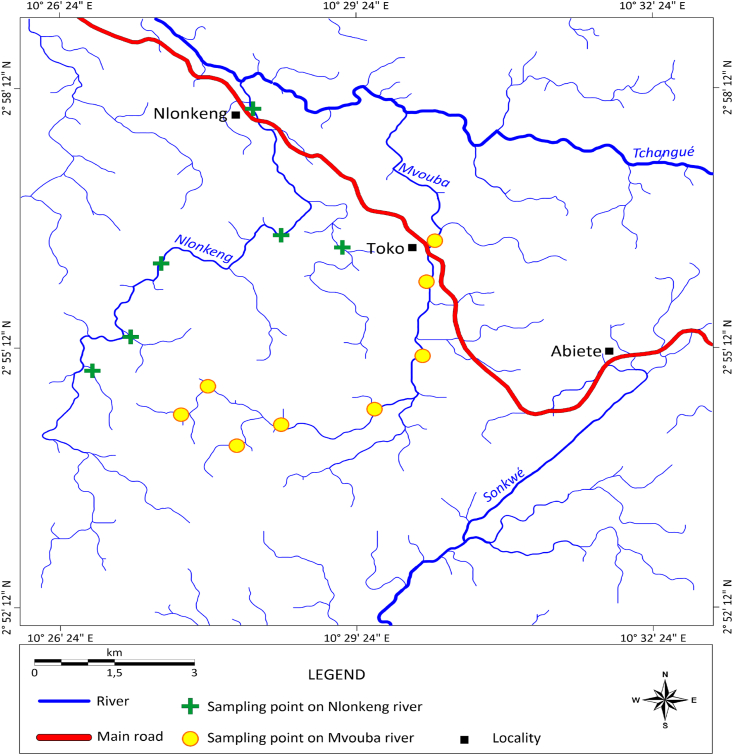
Sample locations map over the drainage pattern.

**Figure 3:**
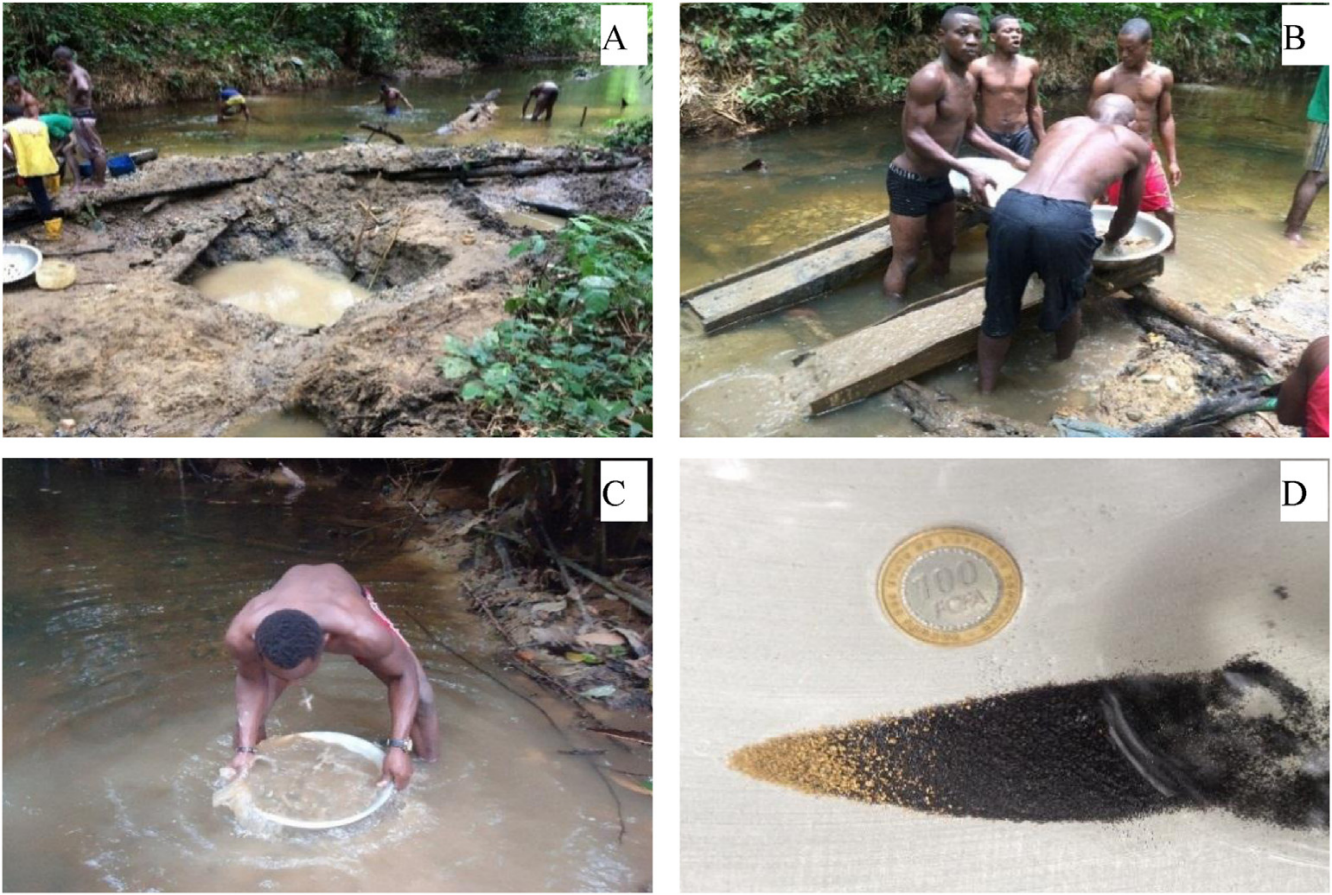
Field Photographs of stream sediment sampling on River Mvouba, A) Sampling point on the river bed, B) Clay and rock fragment removal using traditional sluices, C) panning for of stream sediments for heavy mineral concentration, D) Heavy mineral and gold grains obtained after panning.

The gold grains collected from the Mvouba and Nlonkeng Rivers were randomly panned. The sampling points were chosen on the active river channels based on the evaluation of alluvial deposits. All the sampling points were located in the river bed positions where water flow energy is lowest to allow deposition of heavy mineral including gold grains. Six (06) samples of stream sediments were collected on River Nlonkeng and eight (08) samples on River Mvouba (Figure 2). The geographic coordinates of each sample were recorded using a GPS device. Pits were dug in the river bed to remove the surface sandy material (0 cm to <70 cm depth) in order to expose and to sample the median gravelly zone which is also underlain by a clayey saprolite. The sampled gravel was fed and washed into the traditional sluice. The heavy sand from sluice was later panned manually to concentrate the gold grains (Figure 3). The gold grains obtained were then air-dried and packaged in two polyethylene bags. The bags were then labelled as gold grain Mvouba and gold grain Nlonkeng and sent to Geoscience laboratories (Geo Labs) in Canada for the microchemical and morphological analyses.

In the laboratory, the gold grains were mounted and embedded with peroxide and images were taken to record their morphological characteristics. The quantitative chemical analyses were obtained using a CAMECA SX100 electron microprobe operated at 20 kV-30 nA for major elements and 20 kV-200 nA for trace elements. The elements analysed were Ag, Au, S, Fe, Co, Ni, Cu, Zn, As, Se, Rh, Pd, Pt, Hg and all were detected. The concentrations of the elements in the gold grains enabled to constraint the source rock type. The counting times were set at 20 s or 10 s per element.

## 4 Results

### 4.1 Gold grain morphology

**Figure 4:**
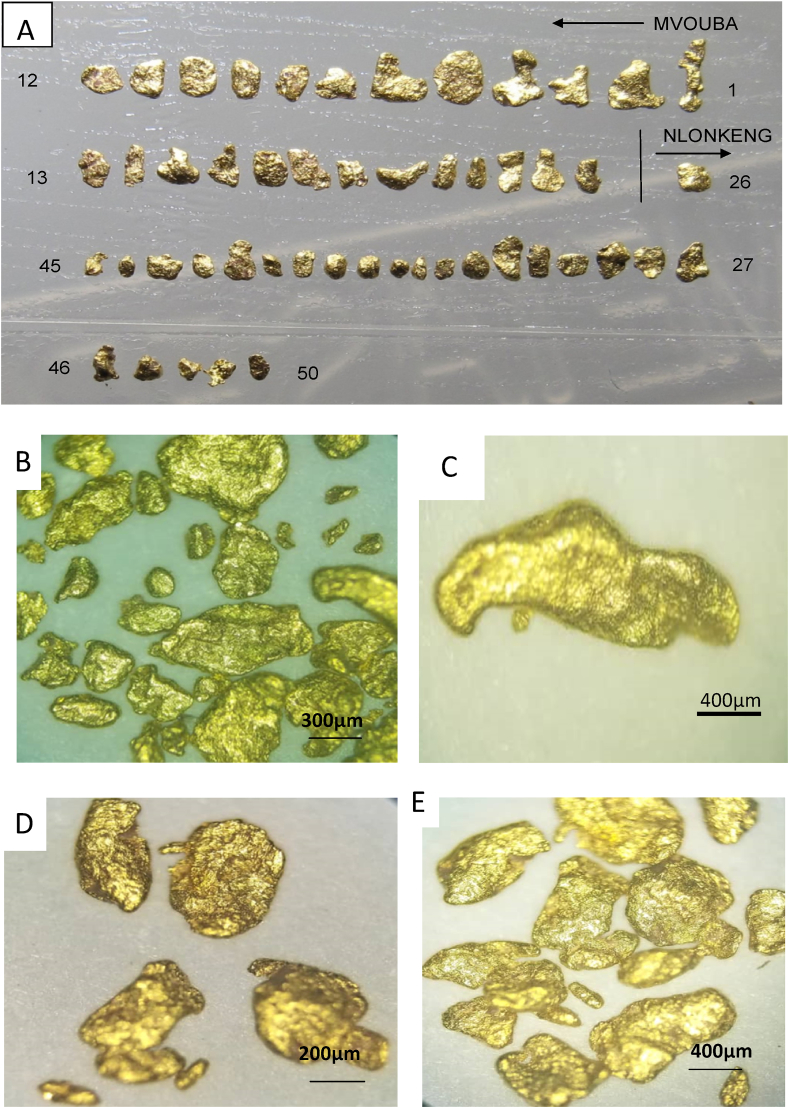
Photographs of grains prior to backfilling with epoxide: Mvouba gold grains; A (labelled 1–25), B and C. Nlonkeng gold grains A (labelled 26–50), D and E. Note the larger irregular grains of Mvouba and the sub-rounded smaller grains of Nlonkeng.

**Figure 5:**
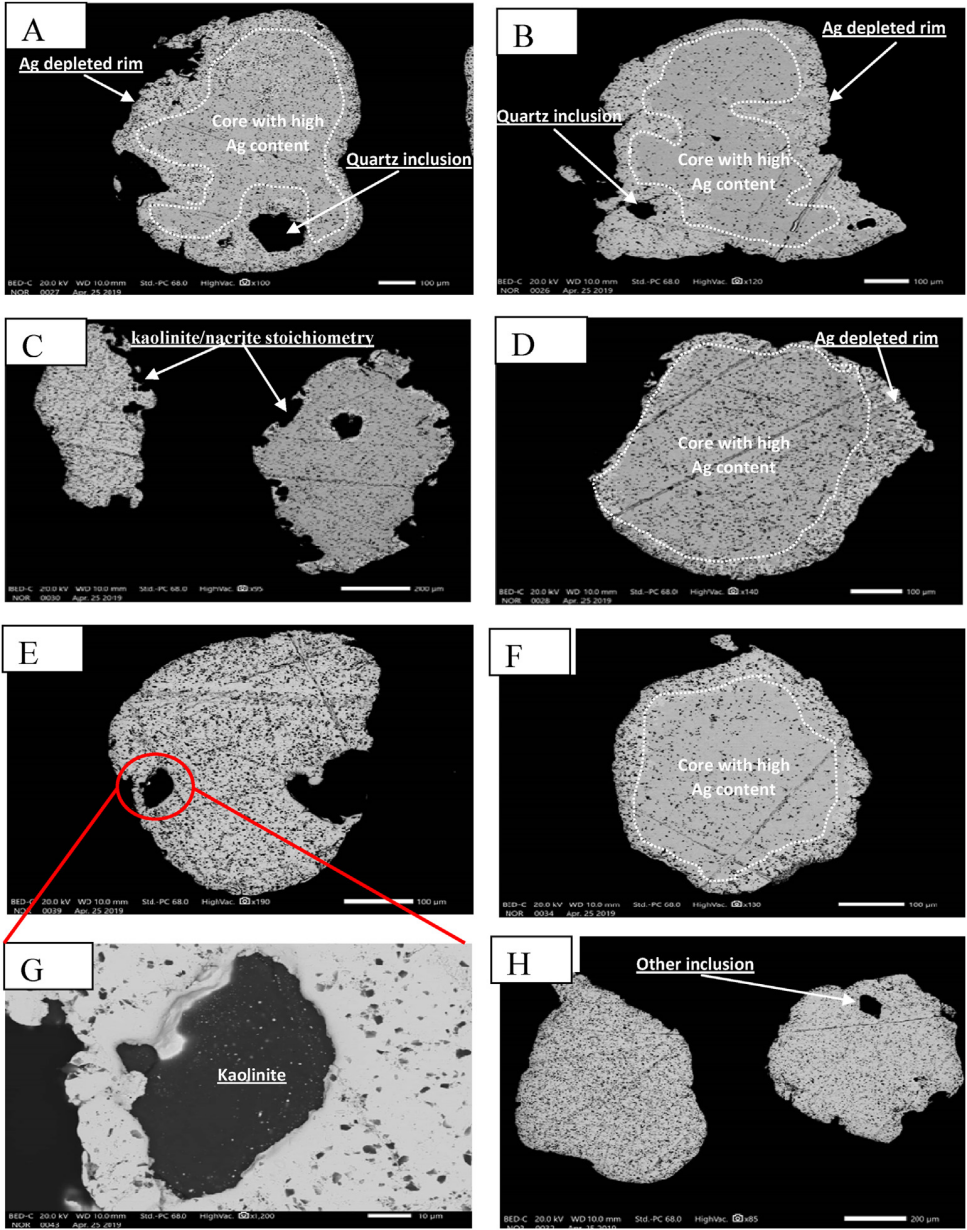
BSE images of gold grains from Mvouba (A, B, C and D) and Nlonkeng (E, F, G and H).

Images of gold grains from Mvouba and Nlonkeng Rivers are shown in Figure 4. They are composed of fine (<1.5 mm) to coarse grains (>1.5 mm). The BSE images (Figure 5) of selected gold grains from Mvouba show relatively larger sizes and the majority of the grains are sub-angular with few elongated to sub-rounded ones. These gold grains also show an irregular outline. Most of the gold grains are zoned with irregular core to rim boundary. Some grains contain inclusions (quartz) and silicate (kaolinite) particles are attached at the rims. Images with darker core zones in some of the grains have high Ag content and the brighter rims have lower Ag and very high Au contents. The BSE images (Figure 5) of Nlonkeng gold grains show smaller grain sizes compared to the Mvouba gold grains and sub-rounded to rounded shapes with fewer elongated to bean-shaped grains. Their outlines are regular. The gold concentration at the rims is lower and they contain numerous quartz inclusions. Images with darker core zones in some of the grains have high Ag contents and the brighter rims have lower Ag and higher Au concentrations.

The gold grains from the Mvouba and Nlonkeng Rivers have diverse morphologies. The morphology of the gold grains from each river enables to know whether they are formed in-situ or have been transported. The Mvouba gold grains have undergone moderate transport distance based on the irregular outline, sub-rounded to elongate shape and variable grain sizes. The Nlonkeng gold grains have undergone longer fluvial transport distances based on their nearly uniform grain size and sub-angular to sub-rounded grains compared to the Mvouba gold grains. Chemical and mechanical weathering of the gold grains might have led to size reduction and rounded shape as they were dislodged from mineralized veins into the weathering/transportation cycle (Suh and Lehmann, 2003).

The Nlonkeng and Mvouba gold grains show Ag-depleted rims and Ag-rich cores probably due to leaching of Ag as the gold grains move down the weathering cycle. This phenomenon is common with gold grains in tropical areas and is often attributed to surficial processes (Groen et al., 1990; Suh and Lehmann, 2003).

### 4.2 Gold grain microchemistry

The quantitative microchemical data of the Mvouba and Nlonkeng gold grains by EMPA is compiled in Tables 1 and 2.

**Table 1:** Electron microprobe data (wt.%) of gold grains from the Mvouba (n = 27).

Sample code	Ag	Au	S	Fe	Co	Ni	Cu	Zn	As	Se	Rh	Pd	Pt	Hg	Total
MG-1	7.65	93.24	0.01	n.d.	n.d.	0.01	0.03	n.d.	n.d.	n.d.	n.d.	n.d.	n.d.	n.d.	100.93
MG-2	4.74	95.17	n.d.	n.d.	0.01	0.02	0.15	n.d.	n.d.	n.d.	0.01	n.d.	n.d.	n.d.	100.09
MG-3	0.28	99.76	n.d.	n.d.	0.01	0.01	0.23	n.d.	n.d.	n.d.	0.01	n.d.	n.d.	n.d.	100.30
MG-4r1	0.24	99.69	0.01	n.d.	0.01	0.01	0.14	n.d.	n.d.	n.d.	0.01	0.05	0.01	n.d.	100.16
MG-4r2	0.32	99.56	0.02	n.d.	n.d.	n.d.	0.15	n.d.	n.d.	n.d.	n.d.	0.06	n.d.	n.d.	100.11
MG-5	0.22	99.70	0.01	n.d.	n.d.	n.d.	0.15	n.d.	n.d.	n.d.	0.01	0.06	n.d.	0.04	100.18
MG-6	1.74	98.15	0.02	n.d.	n.d.	n.d.	0.16	0.01	n.d.	n.d.	n.d.	n.d.	n.d.	n.d.	100.08
MG-7	0.07	100.04	0.02	n.d.	n.d.	0.01	n.d.	n.d.	n.d.	n.d.	0.01	n.d.	n.d.	n.d.	100.15
MG-8	10.88	89.95	0.02	n.d.	n.d.	0.01	0.03	n.d.	n.d.	n.d.	n.d.	n.d.	n.d.	n.d.	100.88
MG-9	0.16	99.95	0.01	n.d.	n.d.	n.d.	0.01	n.d.	n.d.	n.d.	0.01	n.d.	0.01	n.d.	100.15
MG-10	0.71	99.32	0.01	n.d.	n.d.	0.01	0.18	n.d.	n.d.	0.01	n.d.	0.01	0.01	n.d.	100.25
MG-11	11.29	89.48	0.01	n.d.	n.d.	n.d.	0.03	n.d.	n.d.	n.d.	n.d.	n.d.	0.03	0.08	100.92
MG-12	0.32	99.56	0.01	n.d.	n.d.	n.d.	0.11	n.d.	n.d.	n.d.	n.d.	n.d.	n.d.	n.d.	100.01
MG-13	14.33	86.64	0.01	n.d.	n.d.	n.d.	0.01	0.01	n.d.	n.d.	n.d.	n.d.	n.d.	n.d.	101.01
Mg-14	17.45	83.39	n.d.	0.01	n.d.	n.d.	0.02	n.d.	n.d.	n.d.	n.d.	0.02	n.d.	n.d.	100.90
MG-!5	3.55	96.81	0.01	0.01	n.d.	n.d.	0.05	n.d.	n.d.	n.d.	n.d.	n.d.	n.d.	n.d.	100.43
MG-16	1.37	98.99	0.01	n.d.	n.d.	0.01	0.07	n.d.	n.d.	n.d.	n.d.	n.d.	n.d.	n.d.	100.46
MG-17	0.24	99.87	0.01	n.d.	n.d.	0.01	n.d.	n.d.	n.d.	n.d.	n.d.	n.d.	n.d.	0.05	100.18
MG-18	0.17	99.86	0.01	n.d.	0.02	n.d.	0.01	n.d.	n.d.	n.d.	0.01	n.d.	n.d.	0.01	100.09
MG-19	0.43	99.72	0.01	n.d.	n.d.	n.d.	0.03	n.d.	0.01	n.d.	n.d.	n.d.	n.d.	n.d.	100.20
MG-20	5.89	94.60	n.d.	n.d.	n.d.	n.d.	0.04	0.01	n.d.	n.d.	n.d.	n.d.	n.d.	n.d.	100.54
MG-21	0.59	99.22	0.01	0.01	0.01	n.d.	n.d.	n.d.	n.d.	n.d.	n.d.	0.01	n.d.	n.d.	99.86
MG-22	1.11	98.78	0.01	0.01	n.d.	n.d.	0.32	n.d.	n.d.	0.01	n.d.	0.01	0.02	n.d.	100.25
MG-23	4.65	95.13	0.01	n.d.	n.d.	n.d.	0.13	0.01	n.d.	n.d.	n.d.	n.d.	n.d.	n.d.	99.92
MG-24	2.74	97.25	0.01	n.d.	n.d.	n.d.	0.11	n.d.	0.01	n.d.	n.d.	n.d.	n.d.	n.d.	100.11
MG-25	0.46	99.15	0.01	n.d.	n.d.	0.01	0.22	0.01	n.d.	0.01	n.d.	n.d.	n.d.	n.d.	99.86
MG-26	0.40	99.82	0.01	n.d.	n.d.	n.d.	0.03	n.d.	0.01	n.d.	n.d.	n.d.	n.d.	n.d.	100.17

n.d.: not detected.

**Table 2:** Electron microprobe data (wt.%) of gold grains from the Nlonkeng River (n = 26).

Sample code	Ag	Au	S	Fe	Co	Ni	Cu	Zn	As	Se	Rh	Pd	Pt	Hg	Total
NG-1	2.29	97.51	0.01	n.d.	n.d.	0.01	0.15	n.d.	n.d.	n.d.	0.01	n.d.	n.d.	n.d.	99.99
NG-2	0.16	99.69	0.01	0.01	0.01	n.d.	0.09	n.d.	n.d.	n.d.	0.01	n.d.	n.d.	n.d.	99.97
NG-3	3.53	96.61	0.01	n.d.	n.d.	n.d.	0.17	n.d.	0.01	n.d.	n.d.	n.d.	0.03	0.02	100.38
NG-4r1	0.38	99.23	0.01	n.d.	n.d.	n.d.	0.17	0.01	0.01	n.d.	n.d.	0.05	n.d.	n.d.	99.85
NG-4r2	0.37	99.13	0.01	n.d.	n.d.	n.d.	0.16	n.d.	n.d.	n.d.	n.d.	0.04	n.d.	n.d.	99.71
NG-5	0.37	98.36	0.02	n.d.	n.d.	n.d.	0.16	0.01	n.d.	n.d.	n.d.	0.05	n.d.	n.d.	98.95
NG-6	0.38	99.88	0.01	n.d.	n.d.	n.d.	0.01	n.d.	n.d.	n.d.	0.02	n.d.	n.d.	n.d.	100.29
NG-7	1.45	98.56	0.01	n.d.	n.d.	n.d.	0.16	n.d.	0.01	n.d.	n.d.	n.d.	n.d.	n.d.	100.18
NG-8	3.32	96.49	0.02	n.d.	n.d.	0.01	0.15	0.01	n.d.	n.d.	0.01	n.d.	n.d.	n.d.	100.01
NG-9	0.70	99.14	0.01	n.d.	n.d.	n.d.	0.08	n.d.	n.d.	n.d.	n.d.	n.d.	0.03	0.04	100.01
NG-10	0.35	99.18	0.02	n.d.	n.d.	0.01	0.13	0.01	n.d.	n.d.	0.01	n.d.	n.d.	n.d.	99.70
NG-11	0.31	98.91	0.02	0.01	n.d.	0.02	0.62	n.d.	n.d.	n.d.	0.01	n.d.	n.d.	n.d.	99.88
NG-12	0.67	98.04	0.01	0.01	n.d.	n.d.	0.96	n.d.	n.d.	n.d.	n.d.	n.d.	n.d.	n.d.	99.69
NG-13	0.43	99.26	0.02	n.d.	n.d.	n.d.	0.21	n.d.	n.d.	n.d.	0.01	0.01	n.d.	n.d.	99.95
Ng-14	9.37	90.56	0.01	n.d.	n.d.	n.d.	0.83	n.d.	0.01	n.d.	n.d.	0.01	n.d.	n.d.	100.79
NG-!5	3.42	96.48	0.03	n.d.	n.d.	n.d.	0.22	n.d.	n.d.	n.d.	n.d.	n.d.	0.05	n.d.	100.21
NG-16	0.74	99.28	0.01	n.d.	n.d.	n.d.	0.09	n.d.	n.d.	n.d.	n.d.	0.01	n.d.	n.d.	100.14
NG-17	0.14	100.01	0.03	n.d.	n.d.	n.d.	n.d.	n.d.	n.d.	0.01	n.d.	n.d.	0.01	n.d.	100.21
NG-18	0.53	99.65	0.02	n.d.	n.d.	0.01	0.02	0.01	n.d.	n.d.	n.d.	0.01	n.d.	n.d.	100.23
NG-19	0.17	100.10	0.02	0.01	n.d.	n.d.	0.09	n.d.	0.01	0.01	n.d.	n.d.	0.01	0.03	100.43
NG-20	0.97	98.89	0.02	n.d.	n.d.	n.d.	0.08	n.d.	0.01	n.d.	n.d.	n.d.	n.d.	n.d.	99.96
NG-21	4.29	96.47	0.01	n.d.	n.d.	0.01	0.07	n.d.	n.d.	n.d.	n.d.	0.01	n.d.	n.d.	100.87
NG-22	3.39	96.84	0.01	n.d.	n.d.	0.01	n.d.	n.d.	n.d.	0.01	n.d.	0.02	n.d.	n.d.	100.26
NG-23	13.62	87.15	0.01	n.d.	n.d.	0.01	n.d.	n.d.	n.d.	n.d.	0.01	0.01	0.02	n.d.	100.82
NG-24	1.31	98.68	0.01	n.d.	n.d.	n.d.	0.08	n.d.	n.d.	n.d.	0.01	n.d.	0.03	n.d.	100.11
NG-25	4.67	95.28	0.02	n.d.	n.d.	n.d.	0.06	n.d.	n.d.	n.d.	0.01	n.d.	0.02	n.d.	100.06
NG-26	0.62	98.24	0.01	0.01	n.d.	n.d.	0.96	n.d.	n.d.	n.d.	n.d.	n.d.	n.d.	n.d.	100.83

n.d.: not detected.

The gold grains from the two rivers are associated with Ag and minor amounts of Cu, As, S and Se. These gold grains show very low concentrations of Zn, Rh, Pd, Pt, Hg, Fe, Ni, Co. The weight percentage of gold for the Mvouba gold grains ranges from 83.40 to 100 wt.% (Table 1) and from 87.10 to 100 wt.% for the Nlonkeng gold grains (Table 2).

**Figure 6:**
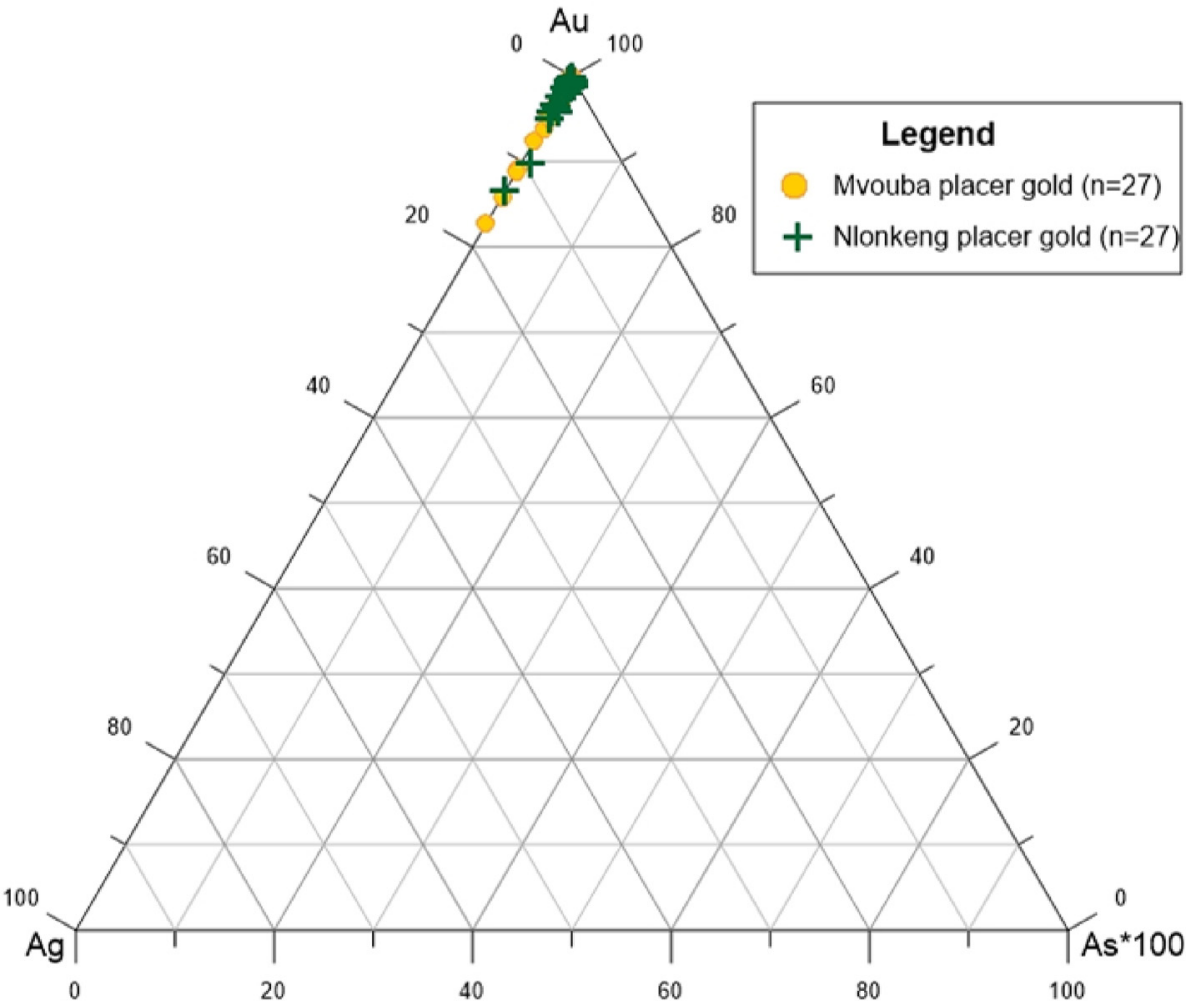
Ternary discrimination plot of Au–Ag–As showing very high content of Au, and extremely low content of As (n = 27).

The Ag content ranges from 0.07 to 17.45 wt.% and from 0.14 to 13.62 wt.% for Mvouba gold grains and Nlonkeng gold grains, respectively. The As concentrations are very low (Figure 6). The Cu content of the Mvouba and Nlonkeng gold grains are above detection limit for almost all the sample but all are <1wt.% (Table 1).

All the gold grains of the two rivers have a significant quantity of quartz inclusions. The fineness of gold grains in these areas ranges from 864 to 1000 for Nlonkeng, and from 826 to 999 for Mvouba.

**Figure 7:**
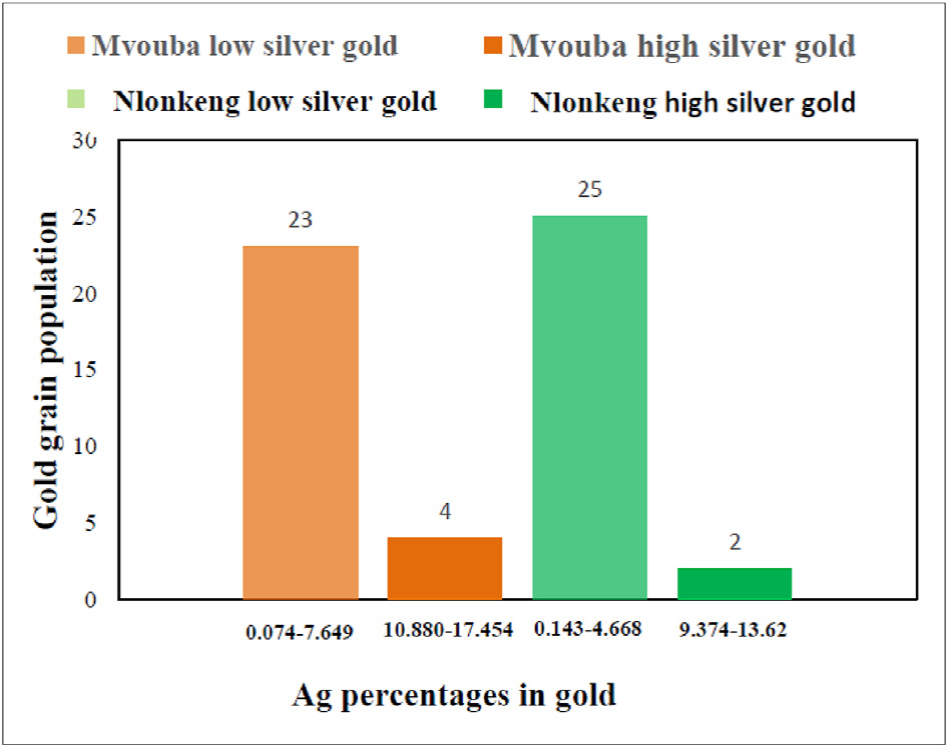
Chart bars exhibiting the two groups of gold grains in Mvouba and Nlonkeng rivers (n = 27).

The gold grains from the two rivers are categorized into low silver gold and high silver gold (Figure 7). According to Wierchowiec (2002), the limit between low- and high silver gold is Ag = 8 wt.%. In Mvouba, 23 grains are low silver-gold (0.07–7.65 Ag wt.%) and only 4 grains are high silver gold (10.88–17.45 Ag wt. %) while 25 grains from Nlonkeng are low silver gold (0.14–4.67 Ag wt. %) and 2 grains are high silver gold (9.37–13.62 wt.%).

## 5 Interpretation and discussion

### 5.1 Gold deposit type

The microchemical signature of gold grains are often used to establish the type of mineralization in different mining districts (Huang et al., 2013). When establishing the microchemical signature of placer gold, the Ag content is the first criterion taken into consideration (Chapman et al., 2017), especially, as the Ag content of gold grains enables to determine the type of deposit (Chapman et al., 2009). The Ag content of mesothermal gold mineralization is lower than that of epithermal gold mineralization (Shikazono and Shimizu, 1988; Chapman and Mortensen, 2006).

The Ag content of Mvouba gold grains and Nlonkeng gold grains, grouped into low (<8 wt.%) and high (>8 wt.%) silver gold, could imply that the grains are from two sources or were formed by two distinct geological processes. The disparity in the Ag content might also be interpreted as a preferential leaching of silver from the gold grains (Stoffregen, 1986; Colin et al., 1989). Therefore, in the Abiete-Toko gold district, gold grains with high Ag wt.% might have experienced little or no leaching and therefore have no external rim, while gold grains with low Ag wt.% show well developed rims and might have been subjected to important leaching after their release from primary source(s).

**Figure 8:**
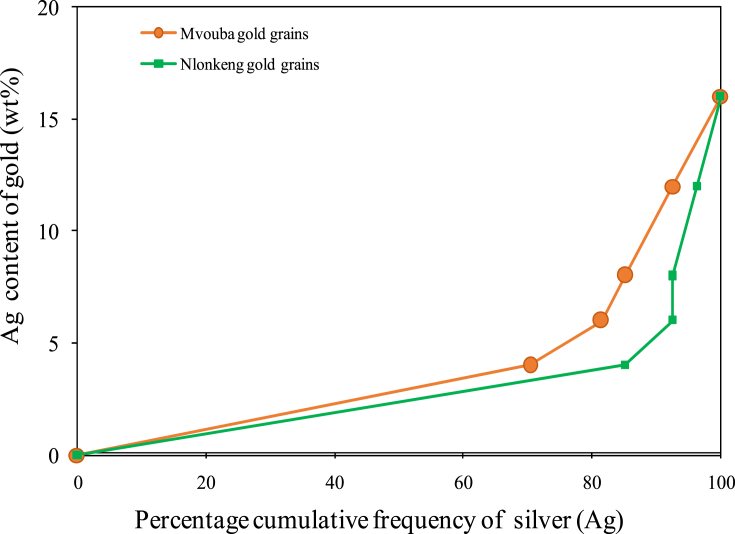
Cumulative plots of Ag contents of Mvouba and Nlonkeng gold grains (n = 27).

Cumulative plots are presented in Figure 8, where gold grains from Mvouba and Nlonkeng Rivers appear in percentile and are plotted against their respective silver contents. These illustrations permit a direct comparison of the studied gold grains and a visual indication of the presence of two populations of gold grains.

Gold fineness is an important criterion used to characterize gold grains (Morrison et al., 1991). Ag is usually leached from the rims of gold grains during transportation in stream sediments leading to an increase in gold fineness with increasing distance from the source (Dumula and Mortensen, 2002). According to Morrison et al. (1991), the fineness values of gold grains differ with different types of gold deposits. The ranges are: 780–1000 for Achaean lode gold deposits; 800–1000 for mesothermal slate belt deposits; 650–970 for plutonic deposits; 650–1000 for porphyry deposits and 0–1000 for epithermal deposits. Gold from mesothermal environments exhibits a narrow range of fineness with average values of 800–1000 and a primary deposition by sulphidation (Morrison et al., 1991). In the present study, the Mvouba and Nlonkeng gold grains have a narrow range of fineness of 826–999 and 864–1000, respectively. The majority of values cluster towards the upper limit closer to the average for Achaean and slate belt gold deposits (920–940) rather than intrusion related deposits (825) (Morrison et al., 1991). The high sulphur contents of the gold could imply that primary deposition of gold was by sulphidation. The high Ni and Co contents in some gold grains could suggest an interaction with greenstones. Low Ag content and high gold fineness values in the studied gold deposits indicate that sulphidation could be the dominant mechanism of ore deposition. Gold grains with Cu contents of less than 1 wt.% could indicate a primary hydrothermal vein type deposit (Vishiti et al., 2015).

The composition of the gold grains from Mvouba and Nlonkeng Rivers were plotted in Au–Ag–Cu∗100 and Au–Ag–Hg∗25 ternary discrimination plots.

**Figure 9:**
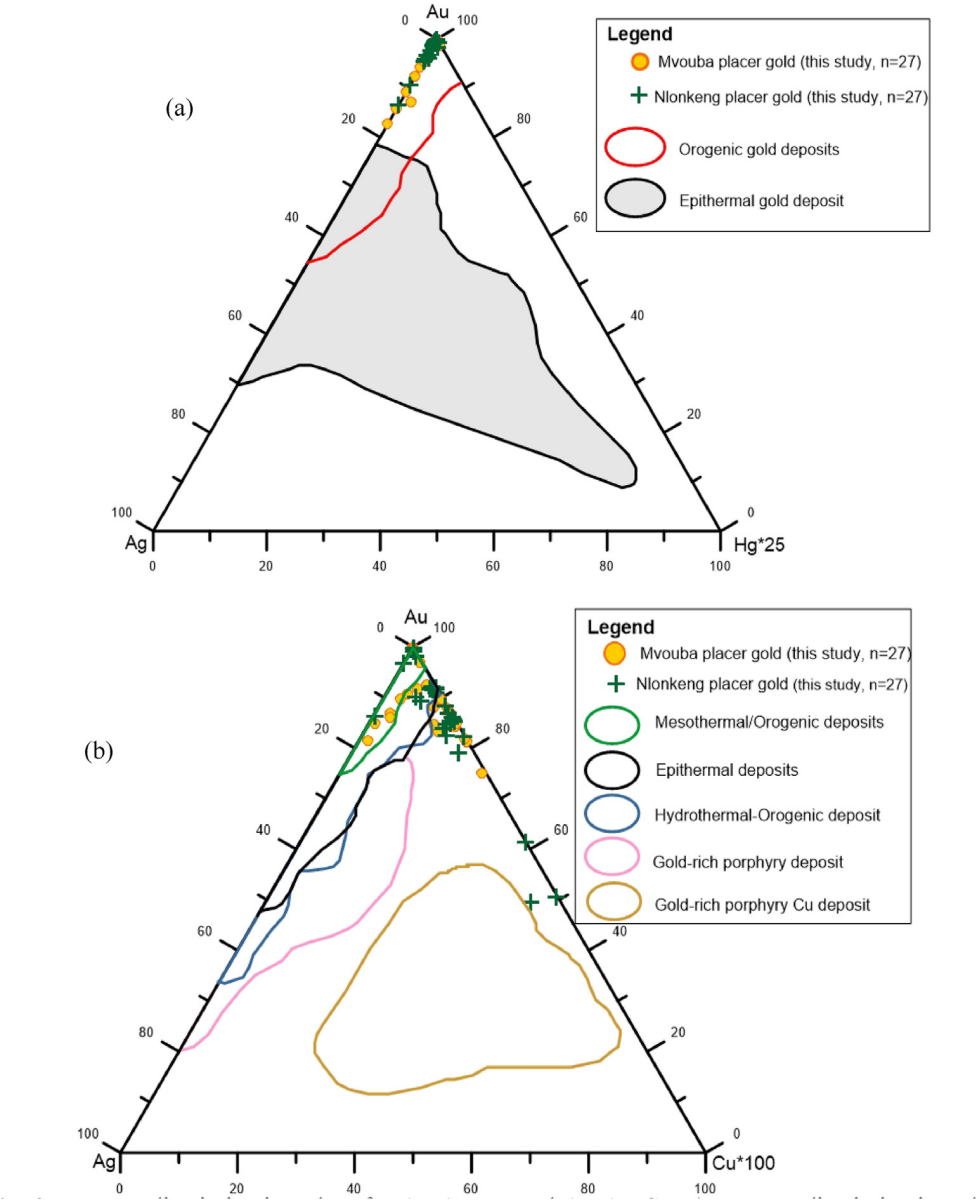
Ternary discrimination plots for Au–Ag–Hg and Au–Ag–Cu. a) Ternary discrimination plot for Au–Ag–Hg with the composition of gold grains from Mvouba-Nlonkeng placer gold. The gold grains in this study falls within the orogenic gold deposits. b) Ternary discrimination plot for Au–Ag–Cu with the composition of gold grains from Mvouba-Nlonkeng placer gold. (n = 27).

Both the Mvouba and Nlonkeng gold grains fall in the zone of orogenic gold deposit (Figure 9). They match with orogenic gold deposits defined by Chapman and Mortensen (2006), Suh et al. (2006), Omang et al. (2015) and Vishiti et al. (2017). This is comparable to low Ag–Au alloys (10–20%Ag) with little or no Hg in the South of Hunker Dome Region (Chapman and Mortensen, 2006) which is a mesothermal gold deposit. Au–Ag–Cu ternary discrimination diagram (Figure 9) for this study yielding Ag<20 wt% and Cu < 1wt%, plots of Mvouba and Nlonkeng river are clustered within the field of epithermal/mesothermal orogenic gold suggesting that the Mvouba and Nlonkeng catchment is an Archean (Eon, Precambrian) mesothermal orogenic gold field.

### 5.2 Implications to gold exploration in Abiete-Toko gold district

Exploration for orogenic gold in the rain forest under tropical climate, where outcrops are rare and weathering profiles are thick, is very challenging. Gold grain morphological characterization from stream sediments stands out as an important exploration tool in orogenic gold localities (Antweiler and Campbell, 1981; Grant et al., 1991; Rubin and Kyle, 1997). This is importance in providing added value to existing exploration methods such as soil sampling, pitting, trenching, mapping…etc (Chapman and Mileham, 2016).

The alloy compositions of gold grains from Mvouba and Nlonkeng Rivers are binary gold-silver, with copper above lower detection limit but <1%. Mercury is almost absent, permitting to infer the Abiete-Toko area as an orogenic gold locality. Assuming this present work is routine stream sediment sampling for an exploration program targeting the lode gold or primary gold deposit in this area where the coarse gold grains are found in almost all streams, geophysical surveys and detailed mapping become the key exploration methods. Magnetic and radiometric methods are the best indirect exploration techniques to identify targets at regional scale, geophysical airborne survey including electromagnetic and gravity is suggested for Abiete-Toko orogenic gold district. According to Robert et al. (2007), gravity is an effective technique for defining the geometry of greenstones belts at regional scale. The majority of world-class orogenic gold deposits are late in the kinematic history of their hosting orogens (Groves et al., 2000) and, therefore, their geometry is commonly preserved making geological maps essential exploration tools. The computer application to gold exploration is of great importance and this enables to generate two-dimensional and three-dimensional maps from geophysical data superimposed to detailed geological maps. This application in Abiete-Toko will yield the targets for drilling exploration. Regional soil sampling, pitting and trenching will not be efficient and are not recommended due to the very thick weathering mantles composed mainly of material transported downslope.

**Figure 10:**
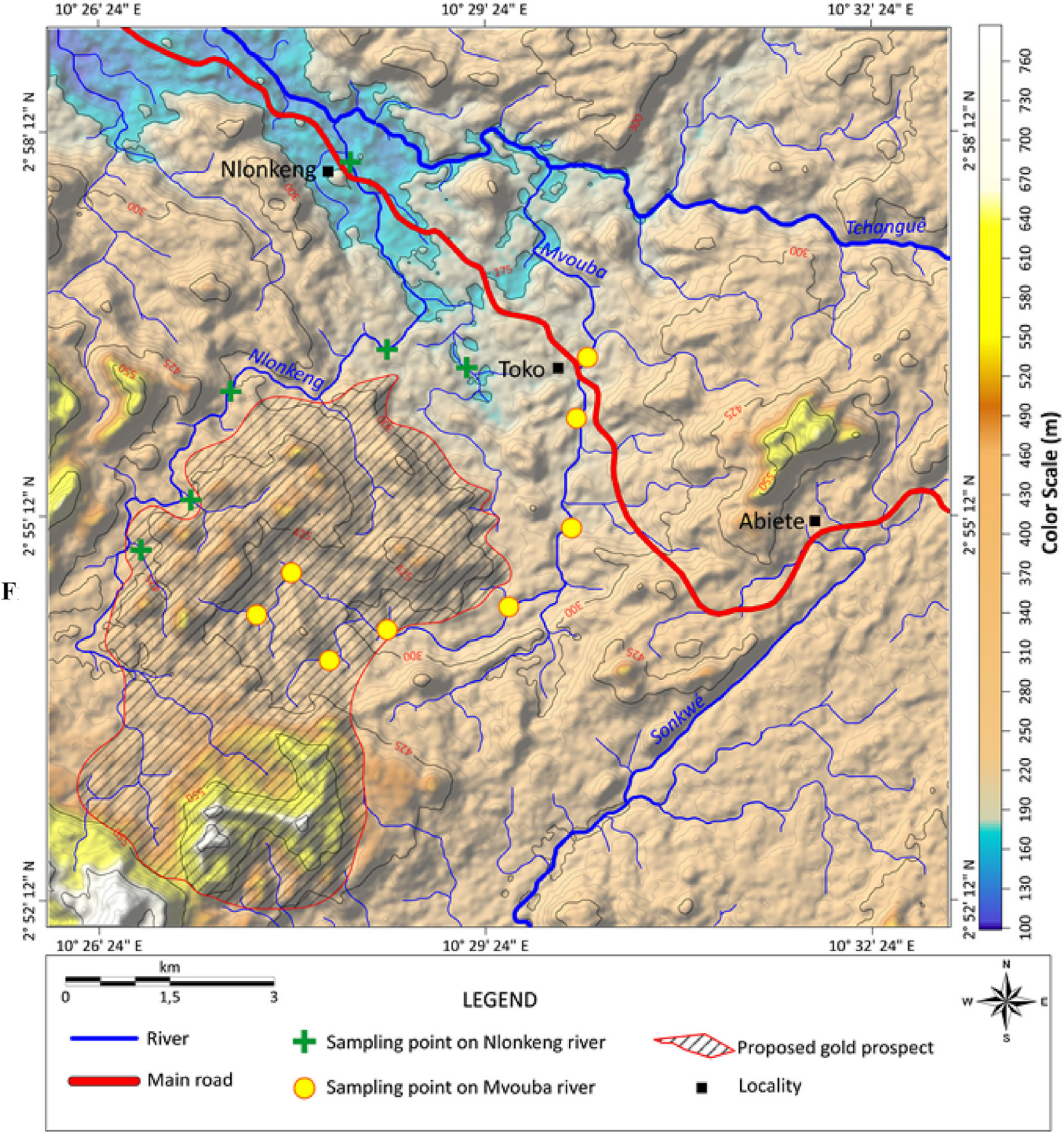
Drainage pattern overlapping the three-dimensional digital elevation model map of Abiete-Toko gold district and highlighting the gold prospect.

Based on geomorphology and the Mvouba-Nlonkeng drainage pattern, a prospect of about 20 km^2^ (Figure 10), where the future exploration work should focus, is proposed. The prospect is made up of a single geomorphological unit where Mvouba and Nlonkeng rivers drain and also rise. The defined prospect is made up of amphibolites, greenstones, felsic gneiss and Banded Iron Formation (BIF), all affected by multiple phases of deformation (Binam Mandeng et al., 2018). The exploration will be targeting the fracture zones and also the contact zones between the BIF, the felsic gneiss and the greenstones as proposed by Shepherd et al. (2005) and Silva et al. (2012) for structure-related orogenic gold mineralization.

## 6 Conclusions and recommendations

The study enables to conclude that:

a) Mvouba and Nlonkeng rivers in Abiete-Toko (South Cameroon) are rich in coarse alluvial gold grains with variable morphologies. The Mvouba gold grains are mostly irregularly shaped with few elongated to sub-rounded grains. The Nlonkeng gold grains are mostly sub-rounded to rounded with few irregularly shaped grains.
b) Mvouba gold grains have undergone moderate fluvial transport distance while the Nlonkeng gold grains have undergone long fluvial transport distance. In river Nlonkeng, there are more gold grains with Ag-leached rims than in river Mvouba.
c) Microchemical studies of gold grains indicate that the gold is alloyed with Ag (<20 wt%) and contains minor amounts of Cu, As, Se, Ni, Co and S. The fineness of gold grains in these areas ranges from 864-1000 for Nlonkeng and 826–999 for Mvouba and therefore the type of deposit is mesothermal/orogenic gold deposit.

It is recommended that:

a) geophysical surveys including gravity, electromagnetic surveys and detailed geological mapping are the key recommended exploration methods to target structures and lithological contacts prior to any drilling program.
b) follow-up stream sediment sampling as well as soil and trench sampling should not be used in Abiete-Toko orogenic gold district due to ubiquitous gold in all streams and tributaries and the very thick weathering mantles mainly made up of transported material.

## Declarations

### Author contribution statement

Gus Djibril Kouankap Nono: Conceived and designed the experiments; Performed the experiments; Analyzed and interpreted the data; Wrote the paper.

Edelquine Fai Bongsiysi: Performed the experiments; Analyzed and interpreted the data; Wrote the paper.

Primus Azinwi Tamfuh, Alexis Jacob Nyangono Abolo, Bertrand Fomekong Kehding, Nicoline Fontem Kibong, Emmanuel Cheo Suh: Conceived and designed the experiments; Performed the experiments; Analyzed and interpreted the data; Wrote the paper.

### Funding statement

Dr Gus Djibril Kouankap Nono was supported by Stones and Gold Sarl.

### Data availability statement

Data will be made available on request.

### Declaration of interests statement

The authors declare no conflict of interest.

### Additional information

No additional information is available for this paper.
